# Developmental care for preterm infants: a scoping review of interventions, outcomes, and implementation contexts

**DOI:** 10.3389/fped.2026.1730571

**Published:** 2026-02-05

**Authors:** Dwi Hastuti, Anggorowati, Zubaidah Zubaidah

**Affiliations:** 1 Doctoral Study Program of Medical and Health Science, Universitas Diponegoro, Semarang, Indonesia; 2 Department of Nursing, Jenderal Achmad Yani University, Cimahi, Indonesia; 3 Department of Nursing, Faculty of Medicine, Universitas Diponegoro, Semarang, Indonesia

**Keywords:** developmental care, family integrated care, kangaroo mother care, neurodevelopment, preterm infants

## Abstract

**Background:**

Preterm infants face high risks of neurodevelopmental, physiological, and psychosocial challenges. Developmental care interventions have become essential strategies to improve infant outcomes and strengthen parental involvement in neonatal intensive care units.

**Objective:**

This systematic review aimed to synthesize evidence on the effectiveness, implementation, and contextual adaptability of developmental care interventions for preterm infants.

**Methods:**

A systematic search of six databases was conducted for studies published between 2020 and 2025. Thirty-five studies met the inclusion criteria, including randomized controlled trials, quasi-experimental, cohort, and mixed-method designs. Interventions analyzed included Family Integrated Care (FICare), Kangaroo Mother Care (KMC), cue-based care, individualized developmental programs, and environmental modifications.

**Results:**

FICare showed consistent benefits in breastfeeding, growth, neurodevelopment, and parental outcomes. KMC improved thermoregulation, cerebral perfusion, and behavioral responses. Cue-based and individualized care enhanced long-term cognitive and motor development, while environmental interventions yielded varied results. Contextual factors—such as parental literacy, infrastructure, and cultural norms—were key to implementation success. Evidence from LMICs emphasized the value of culturally adapted, digitally supported care models.

**Conclusions:**

Developmental care interventions significantly improve outcomes for preterm infants and families. Future research should focus on standardizing outcomes, ensuring long-term follow-up, and applying implementation frameworks to develop scalable, context-specific care models.

## Introduction

Preterm birth is a major contributor to global child morbidity and mortality, affecting approximately 13.4 million newborns each year and accounting for nearly one million deaths globally (1). These infants are at heightened risk for a wide range of adverse outcomes, including neurodevelopmental delay, respiratory dysfunction, gastrointestinal complications, sensory impairments, and cognitive and behavioral disorders (2, 3). The consequences of preterm birth often persist throughout childhood and adulthood, imposing long-term burdens on families, health systems, and national economies, particularly in low- and middle-income countries (LMICs) where access to specialized neonatal care is often limited (4).

**Figure 1:**
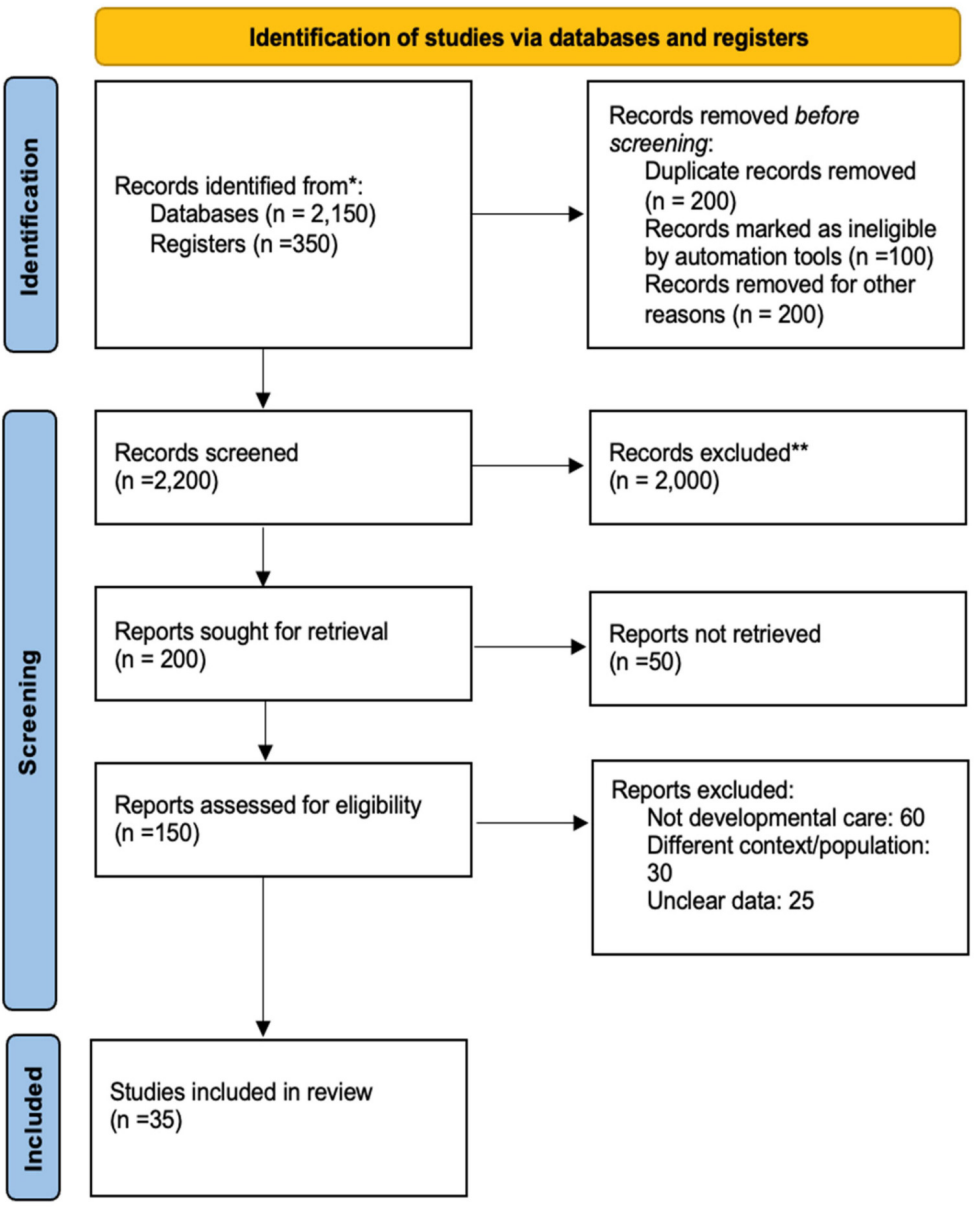
PRISMA flow diagram.

In response to these risks, a shift has occurred in neonatal care toward developmentally supportive, individualized, and neuroprotective approaches. Referred to collectively as developmental care, this model prioritizes interventions aligned with an infant's behavioral cues, physiological status, and neurodevelopmental needs (5). Unlike traditional biomedical frameworks that emphasize survival and clinical stabilization, developmental care draws upon principles of neuroscience, behavioral science, and family systems theory to holistically promote infant well-being within the neonatal intensive care unit (NICU) (6). It reframes the preterm neonate as an active, responsive participant in care; one whose developmental trajectory can be shaped by the environment and the quality of interaction with caregivers (7).

Core components of developmental care include modification of the NICU environment to reduce stressors such as bright light, loud noise, and frequent handling factors shown to disrupt sleep and hinder brain development (8). Skin-to-skin contact, particularly through kangaroo mother care (KMC), has been linked to improved thermoregulation, heart rate variability, weight gain, and early bonding (1, 9). Other elements include therapeutic positioning, which supports postural development and reduces neuromuscular complications (10); cue-based feeding, which fosters gastrointestinal stability and oral-motor coordination (11); and structured family engagement, which empowers parents, reduces anxiety, and promotes shared decision-making (12).

These interventions have been associated with a broad range of clinical, developmental, and psychosocial benefits. Improved cardiorespiratory stability, neurobehavioral outcomes, and shortened hospital stays have been widely reported (13, 14). Additional evidence points to enhanced feeding efficiency, reduced pain response, decreased risk of hospital-acquired infections, and better growth parameters (15–17). From a family systems perspective, family-integrated care models have demonstrated improvements in parental self-efficacy, mental health, and infant–caregiver attachment, which are foundational for long-term socio-emotional development (12, 18, 19).

Despite this growing evidence base, the field remains fragmented in several critical dimensions. First, the literature largely focuses on discrete interventions, such as KMC, sensory modulation, or parental presence, without integrating them into a comprehensive, systems-based framework that reflects the complexity of real-world NICU environments (20). Second, outcome measures vary considerably, with studies reporting inconsistent metrics ranging from physiological parameters to psychosocial indicators, limiting comparability and evidence synthesis (15, 16). Third, although developmental care has been increasingly embedded into neonatal protocols in high-income settings, its adoption and evaluation in LMICs remain limited, inconsistent, and poorly documented (2). Implementation barriers such as workforce shortages, cultural norms, parental literacy, and infrastructural constraints further complicate equitable delivery in resource-constrained settings (19, 21). Additionally, previous reviews have tended to concentrate on individual modalities or narrowly defined clinical outcomes, offering limited insight into how various components of developmental care interact synergistically or are adapted across diverse healthcare contexts (4, 22). There is a notable lack of synthesis regarding how contextual factors, such as NICU layout, staffing models, or sociocultural influences shape implementation fidelity, feasibility, and sustainability. This leaves a significant knowledge gap concerning how developmental care functions as a system-level innovation, particularly in under-resourced environments where neonatal outcomes are most vulnerable.

This scoping review seeks to address these critical gaps by systematically mapping the breadth and diversity of evidence on developmental care for preterm infants. Specifically, it explores: (1) the range of developmental care interventions described in the literature, (2) the outcomes these interventions aim to influence, and (3) the contextual factors that support or hinder their implementation across different health systems. By synthesizing what is currently known and identifying gaps in research and practice, this review aims to inform future studies, guide the development of context-sensitive clinical protocols, and support global policy efforts to expand equitable access to high-quality, developmentally appropriate neonatal care.

## 2 Methods

This scoping review was conducted using the methodological framework proposed by Arksey and O'Malley (23), refined by Levac et al. (24), and guided by the PRISMA-ScR checklist (25). This approach enables comprehensive mapping of evidence related to developmental care interventions for preterm infants, their outcomes, and implementation contexts across healthcare settings.

### 2.1 Eligibility criteria

We included peer-reviewed primary studies that met the following criteria: (1) Population: Preterm infants (born before 37 weeks of gestation). (2) Concept: Developmental care interventions, including but not limited to kangaroo mother care, noise/light reduction strategies, individualized developmental support, family-integrated care, and neuroprotective practices. (3) Context: Neonatal intensive care units (NICUs), step-down units, and community-based follow-up care. (4) Study Types: Quantitative (RCTs, cohort, cross-sectional), qualitative, and mixed-methods studies were included. Reviews, editorials, conference abstracts, and protocol papers were excluded. Only studies published in English between January 2020 and July 2025 were included.

### 2.2 Information sources and search strategy

A comprehensive and systematic literature search was conducted across five major electronic databases: PubMed, Scopus, CINAHL, Web of Science, and EMBASE. These databases were selected to capture a wide range of interdisciplinary studies spanning medicine, nursing, and public health. To ensure thoroughness and reduce publication bias, supplementary searches were performed through Google Scholar and manual screening of the reference lists of all included studies to identify additional relevant literature that may not have been indexed in the primary databases. The search strategy was developed in consultation with a health sciences librarian and involved a combination of controlled vocabulary (e.g., MeSH terms) and free-text keywords. The terms were iteratively tested and refined to maximize sensitivity and specificity. Key search terms included: (“preterm infant” OR “premature baby” OR “low birth weight infant”) AND (“developmental care” OR “kangaroo care” OR “neonatal individualized care” OR “family-centered care”) AND (“intervention” OR “program” OR “strategy”). Boolean operators (AND, OR), truncation symbols, and database-specific filters (e.g., language, publication year) were applied to tailor the search to each platform.

The bibliographic search was conducted between 1 July and 15 August 2025 across multiple databases. The search strategy was designed to identify recent and high-quality evidence; therefore, only peer-reviewed articles published in English between January 2020 and July 2025 were included. The search targeted titles and abstracts using predefined keywords and Boolean operators to capture studies focusing on developmental care for preterm infants. Whenever full texts were available, they were retrieved and screened in full to confirm eligibility, assess methodological quality, and extract detailed data. All search results were documented systematically, including the number of records retrieved, screened, excluded, and retained, following PRISMA-ScR guidelines. A reference management system was used to remove duplicates and maintain an auditable trail of decisions during the screening and selection process, ensuring the reproducibility and transparency of the review.

### 2.3 Selection of sources of evidence

All search results were first imported into EndNote 20 for reference management, where duplicate records were systematically identified and removed. Following this, two reviewers independently screened the titles and abstracts of the remaining studies to assess their relevance according to the predefined eligibility criteria. For studies that appeared to meet the inclusion criteria or where eligibility was unclear, the full texts were retrieved for further evaluation. These full-text articles were then independently assessed by the same two reviewers to determine their final inclusion in the review. In instances where discrepancies arose between the reviewers regarding the eligibility of specific studies, consensus was achieved through discussion. If agreement could not be reached, a third reviewer was consulted to provide resolution and ensure methodological rigor.

### 2.4 Data charting process

A standardized data extraction form was developed and pilot-tested to ensure consistency and reliability in capturing relevant information across studies. Using this form, two reviewers independently extracted data from each included article. Extracted variables encompassed the following elements: author(s), year of publication, and country of origin; study objectives and methodological design; characteristics of the preterm infant population (including gestational age, birth weight, and clinical status); type and detailed description of the developmental care intervention implemented; outcome domains assessed, including neurodevelopmental, behavioral, physiological, and parental outcomes; setting and context of intervention implementation (e.g., NICU level, healthcare system characteristics, or geographic setting); and key findings, including reported effects, feasibility, and limitations of the intervention. Any discrepancies in data extraction were resolved through discussion, with input from a third reviewer when necessary to ensure accuracy and consensus.

### 2.5 Quality appraisal

Although quality assessment is not mandatory in scoping reviews, a structured critical appraisal was conducted to enhance the interpretability and contextual relevance of the included studies. The Mixed Methods Appraisal Tool (MMAT) 2018 version was employed to assess the methodological quality of all eligible empirical studies, encompassing randomized controlled trials (RCTs), non-randomized studies, qualitative studies, mixed-methods designs, and quantitative descriptive studies (26). Each study was first categorized according to its methodological design. Two independent reviewers (initials blinded for peer review) evaluated each study against five design-specific criteria in the MMAT. Responses were rated as “Yes,” “No,” or “Can't tell” for each criterion. Discrepancies were resolved through discussion or adjudicated by a third reviewer. The appraisal focused on internal validity, clarity of research questions, adequacy of data collection and analysis methods, and coherence between findings and conclusions. No studies were excluded based on quality scores; instead, appraisal results were used to contextualize evidence strength across themes and are summarized narratively in the results section (Supplementary Material S1).

Inter-rater agreement was calculated using Cohen's kappa coefficient to ensure consistency of ratings. Studies with lower MMAT ratings were interpreted cautiously in the synthesis phase, especially when drawing conclusions about effectiveness, feasibility, or transferability of developmental care interventions.

### 2.6 Synthesis of results

Extracted data were synthesized using a descriptive approach to provide an overview of the existing evidence. A combination of narrative and thematic synthesis was employed to systematically map the breadth and diversity of developmental care interventions, the associated outcome domains, and the contextual factors influencing implementation, such as geographic location, healthcare setting, and resource availability. The findings were organized to highlight patterns, similarities, and variations across studies. To enhance interpretability, the results are presented in tabular format and thematically grouped according to the type of intervention, targeted outcomes (e.g., neurodevelopmental, physiological, psychosocial), and contextual elements. This method allowed for the identification of gaps in the literature and emerging trends in the delivery of developmental care for preterm infants.

## 3 Results

### 3.1 Searching results

To enhance transparency in study selection, the PRISMA 2020 flow diagram was supplemented with detailed documentation of each exclusion step. An initial 2,500 records were identified across databases and registers. Prior to screening, 500 records were removed through automated and manual filtering. Automated duplicate identification using EndNote 21.2 removed 200 duplicates, while Rayyan's machine-learning relevance classifier (2024 Beta version) flagged 100 records as ineligible based on population mismatch, document type, or methodological irrelevance; all were manually confirmed before exclusion. An additional 200 records were removed for other reasons, including non-English publications (*n* = 72), publication dates outside the 2020–2025 range (*n* = 55), lack of abstracts (*n* = 32), non–peer-reviewed material (*n* = 28), and retracted publications (*n* = 13).

A total of 2,200 records were screened manually at title and abstract level by two independent reviewers, resulting in 2,000 exclusions due to misalignment with inclusion criteria. Full text was sought for 200 records; however, 50 articles could not be retrieved despite attempts via institutional libraries, interlibrary loans, author contact, and academic networks. All 50 abstracts were examined, and while some appeared potentially relevant, lack of methodological detail prevented reliable inclusion. Among the 150 full-text articles assessed, 115 were excluded due to irrelevance to developmental care (*n* = 60), different populations (*n* = 30), or insufficient data (*n* = 25). Ultimately, 35 studies met all criteria and were included in the final synthesis (Figure 1).

### 3.2 Quality appraisal

Studies employing randomized controlled trial (RCT) designs represented the highest methodological quality in this review. For example, Arslan et al. (15), Çaka et al. (16), Wang et al. (17), and Tiryaki et al. (21) clearly articulated randomization procedures, used control groups, and reported outcomes with minimal bias, thereby supporting strong causal inferences regarding the effectiveness of Kangaroo Mother Care (KMC) and Family Integrated Care (FICare). These trials consistently showed improvements in physiological stability, neurobehavior, and caregiver preparedness.

High-quality cluster RCTs and prospective cohort studies such as those by Murphy et al. (27) and Moe et al. (28) offered robust longitudinal data linking FICare interventions to improved breastfeeding rates, neurodevelopmental scores, and reduced hospital stay durations. These studies demonstrated adequate control for confounding variables and high follow-up retention, enhancing confidence in the reliability of their findings. Moderate-to-high quality quasi-experimental studies and mixed-methods research, including work by Chaudhari et al. (29), Kostilainen et al. (30), and McFadden et al. (31), contributed valuable evidence on the psychosocial and developmental effects of maternal-infant bonding interventions, such as maternal singing during KMC and cue-based feeding. Although these studies lacked randomization or full blinding, they employed rigorous outcome measurement tools and added contextual insights regarding intervention feasibility and acceptability.

Retrospective and non-randomized studies such as Liang et al. (32), Zahedpasha et al. (6), and Yang et al. (33) were rated as moderate quality due to design limitations, including potential selection bias and limited adjustment for confounders. Nevertheless, they offered important contributions, particularly in underrepresented settings like China and Iran, by documenting long-term effects of FICare on language development, growth, and microbiome composition. Smaller feasibility and pilot studies, including Rivera (34) and Menke et al. (7), were rated as moderate to low quality due to small sample sizes, absence of comparison groups, and short follow-up periods. However, they provided innovation-focused insights into caregiver education, music therapy, and other adjunctive developmental care modalities.

Despite the variation in design rigor, most studies clearly articulated research objectives and aligned their methods accordingly. However, consistent gaps included the underuse of implementation science frameworks, insufficient blinding, and limited long-term tracking. These findings underscore the need for more methodologically integrated developmental care research, particularly in low- and middle-income countries (20). Overall, the included studies provide a reasonably strong evidence base, with opportunities to strengthen quality through more rigorous trial designs and implementation-focused methodologies (Supplementary Material S1).

### 3.3 Narrative summary of included studies

This systematic review includes 35 primary studies investigating various developmental care interventions for preterm infants. These studies collectively reflect diverse geographic regions, research designs, clinical settings, intervention modalities, and outcome domains, offering comprehensive insight into the global implementation of developmental care strategies in neonatal intensive care units (NICUs) (Table 1).

**Table 1:** Characteristics of included studies and summary of findings.

Authors (Year)	Country	Study design	Setting	GA/BW/clinical status	Intervention	Outcomes	Key findings
(12)	Netherlands	Stepped-wedge cluster RCT	Six NICUs	Preterm infants transferred post-acute phase	Family Integrated Care (FICare) post-transfer	Parental stress (PSS:NICU)	FICare reduced stress in transferred parents but not uniformly across families.
(15)	Turkey	RCT	NICU	Late preterm infants	Kangaroo Mother Care	Cerebral oxygenation, comfort, physiological parameters	Improved cerebral oxygenation and comfort
(16)	Turkey	RCT	NICU	Preterm infants	Kangaroo Mother Care	Feeding intolerance	KMC reduced feeding intolerance
(29)	India	Quasi-experimental	NICU	Mean GA: 33 ± 1.7 wks; BW: 1,698 ± 495 g; stable preterms	Kangaroo Mother Care (KMC)	Cerebral blood flow, vital signs	KMC improved cerebral perfusion and vital stability; benefits sustained post-intervention
(18)	Canada	Quasi-experimental	NICU, follow-up	Very preterm infants	FICare	Behavioral and neurodevelopmental outcomes at 18 months	Infants exposed to FICare showed improved self-regulation and behavioral outcomes
(22)	Spain	RCT	NICU	Preterm infants and mothers	Kangaroo Mother Care	Physiological stress markers	KMC reduced cortisol levels and stabilized HR in both mothers and infants.
(13)	Egypt	Randomized Controlled Trial	NICU, Tertiary Hospital	GA < 34 weeks; clinically stable preterm	Prolonged Kangaroo Care (3 hours/day for 6 days) vs. short KC	Neurobehavior (NBAS), Feeding performance (POFRAS)	Longer KC significantly improved neurobehavioral outcomes and feeding performance.
(46)	Japan	Quasi-experimental	NICU	Preterm infants	Kangaroo Mother Care	Chromogranin A, perfusion index	KMC influenced stress biomarkers and perfusion
(35)	Turkey	RCT	NICU	Preterm infants; mothers postpartum	Intermittent Kangaroo Care	Maternal attachment, postpartum depression	Improved attachment and reduced depression
(38)	USA	Descriptive study	NICU	Extremely preterm infants	Small Baby Unit (SBU) Model	Neonatal outcomes	Improved care outcomes for extremely preterm infants
(45)	Egypt	Quasi-experimental	NICU	Preterm infants (GA not specified)	Healing environment and clustered nursing care	Vital signs, pain, sleep duration	Significant improvement in sleep, reduced pain, and stable vital signs in intervention group.
(43)	China	Experimental Study	NICU	Preterm infants	Developmental care + Kangaroo care	Neurological development, immune function	Combination improved neurodevelopment and immune indicators more than standard care.
(30)	Finland	Mixed-methods	NICU	Mothers with preterm infants	Maternal singing during kangaroo care	Maternal anxiety, well-being, bonding	Singing reduced maternal anxiety and enhanced bonding experience with infants.
(36)	Turkey	Quasi-experimental	NICU	Preterm neonates	Kangaroo Care	Breastfeeding rate, neurodevelopment	Kangaroo care enhanced breastfeeding and improved neurodevelopmental outcomes.
(32)	China	Retrospective cohort	NICU	Premature infants	FICare	Growth, language development at 18 months	Infants in FICare group had significantly better growth and language development scores
(31)	UK	Mixed-methods feasibility study	NICU	Preterm infants (GA < 34 weeks)	Cue-based vs. scheduled feeding	Feasibility, feeding efficiency	Cue-based feeding feasible and promising; further trials needed.
(7)	Germany	Pilot study	NICU	Preterm infants and caregivers	Family-centered music therapy	Parental empowerment, infant regulation	Music therapy empowered parents, enhanced infant regulation and bonding.
(28)	Canada	Prospective cohort	NICU (Alberta FICare)	Preterm infants (GA <34 weeks)	Family Integrated Care (FICare)	Neurodevelopment, behavior, growth (2–24 months)	Improved neurodevelopmental scores and growth in FICare group, especially at 6–24 months.
(39)	USA	Prospective Study	NICU	Preterm infants on bubble CPAP	Cue-based feeding	Time to full oral feeding	Cue-based feeding accelerated transition to full oral feeding in infants on respiratory support.
(19)	Spain	Case-control	Level IIIC NICU	High-risk preterms	FICare	Morbidity and length of stay	FICare associated with reduced neonatal morbidity and shorter hospital stays
(27)	Canada	Cluster-RCT	Level II NICUs	GA: 32–34 + 6 wks; VLBW	Alberta FICare	Neonatal outcomes (feeding, weight, LOS)	FICare improved breastfeeding rates, weight gain, and reduced hospital length of stay
(41)	USA (Multi-site)	Longitudinal cohort	Multiple NICUs (ECHO program)	Very preterm infants	Environmental factors assessment	Developmental, health, and psychosocial outcomes	Environmental exposures significantly impact long-term developmental outcomes in very preterm infants.
(14)	Ukraine	Prospective Cohort	NICU	Very preterm infants	Kangaroo Mother Care (KMC)	Short-term neonatal outcomes	KMC improved thermoregulation, oxygenation, and weight gain in very preterm neonates.
(34)	USA	Program Development	NICU	NICU caregivers of preterm infants	Cue-based feeding education	Caregiver self-efficacy	Education program enhanced caregivers’ self-efficacy in implementing cue-based feeding.
(47)	Iran	Non-randomized trial	NICU	Preterm infants <34 weeks	Cue-based feeding	Feeding intolerance, length of stay, weight gain	Cue-based feeding improved tolerance and reduced hospital stay.
(42)	India	Comparative Study	NICU	LBW preterm neonates	Cue-based vs. scheduled feeding	Feeding efficiency, weight gain	Cue-based feeding resulted in better weight gain and feeding tolerance than scheduled feeding.
(48)	Brazil	Observational Study	NICU	Preterm infants and mothers	Kangaroo Care	Chronic stress response	Kangaroo care modulated stress response in both mothers and preterm infants.
(40)	USA	Quality Improvement Study	NICU	Preterm infants	Cue-based feeding	Feeding readiness, parental involvement	Improved infant feeding outcomes and increased parental confidence and participation in care.
(21)	Turkey	RCT	NICU	Preterm infants	FICare	Parental preparedness for discharge	FICare significantly enhanced parental readiness and feeding practices before discharge
(11)	Netherlands	Retrospective Cohort + Mediation Analysis	Single Family Rooms in NICU	Preterm infants	FICare in Single Family Rooms	Late-onset sepsis (LOS)	FICare reduced LOS; effect mediated by increased parental presence.
(44)	India	Pre-post	NICU	Preterm neonates	KMC post-immersion bath	Body temperature stabilization	KMC post-bath helped maintain temperature
(17)	China	RCT	NICU	Preterm infants	KMC	aEEG activity, neurobehavior	KMC improved neurobehavior and brain activity
(33)	China	Preliminary Comparative Study	NICU	Preterm infants with NEC and enterostomy	FICare vs. standard care	Intestinal microbiome composition	FICare associated with more diverse and beneficial gut microbiota.
(37)	Turkey	Crossover study	NICU	Premature infants	KMC by both parents	HR, oxygen saturation, comfort levels	KMC by both parents yielded better outcomes
(6)	Iran	Non-randomized trial	NICU	Preterm infants	Cue-based feeding	Feeding performance, weight gain	Enhanced weight gain and feeding coordination observed.

GA, gestational age; BW, birth weight; VLBW, very low birth weight; LOS, length of stay; FICare, family integrated care; KMC, kangaroo mother care; NIDCAP, Neonatal Individualized Developmental Care and Assessment Program.

#### 3.3.1 Geographic distribution and study settings

The studies were geographically diverse, with the highest number conducted in Turkey (15, 16, 21, 35–37), followed by the United States (34, 38–42), and China (17, 32, 33, 43). Other countries represented include Canada (18, 27, 28), India (42, 44), Egypt (13, 45), as well as Spain, Netherlands, Japan, Iran, Brazil, Finland, Ukraine, Germany, and the UK. The majority of studies were conducted in tertiary-level NICUs, highlighting the clinical intensity and complexity associated with caring for preterm infants in such settings.

#### 3.3.2 Study designs and methodological approaches

Among the 35 included studies, randomized controlled trials (RCTs) constituted the largest group (*n* = 7) (13, 15–17, 21, 22, 35). In addition, the review included four quasi-experimental studies (29, 36, 45–47), two cohort studies (14, 28), and one observational study (48). The evidence base also incorporated two mixed-methods studies and one feasibility or pilot study, reflecting a growing trend toward integrating quantitative and qualitative approaches to developmental care research (30, 31).

#### 3.3.3 Population characteristics

All studies targeted preterm infants, with gestational ages ranging from extremely preterm (<28 weeks) to late preterm (34–36 weeks). Many focused on clinically stable infants (13, 29) or those on specific interventions like CPAP (39). Several studies incorporated maternal variables and psychological outcomes (30, 35), underscoring the importance of mother-infant dyads in developmental care interventions.

### 3.4 Thematic findings

The present scoping review identified six major thematic domains in the current evidence base regarding developmental care interventions for preterm infants in NICU settings: (1) Family-Centered and Integrated Care, (2) Kangaroo Mother Care (KMC), (3) Cue-Based and Individualized Developmental Care, (4) Environmental Optimization, (5) Multisensory and Psychosocial Interventions, and (6) Contextual and Implementation Factors (Table 2).

**Table 2:** Thematic interpretation of developmental care interventions in NICU settings.

Thematic domain	Subthemes	Interventions	Key outcomes	Interpretation/Implication
Family-centered and integrated care	- Family presence - Caregiver empowerment - Shared decision-making	Family integrated care (FICare), Alberta FICare	↑ Breastfeeding rates, ↓ length of stay, ↑ parental discharge readiness, ↓ parental stress, ↑ neurodevelopmental scores	Involving parents as care partners leads to better neonatal and psychosocial outcomes. Effective across high- and middle-income countries with potential for digital enhancement.
Kangaroo Mother Care (KMC)	- Skin-to-skin contact - Physiological stability - Stress reduction - Bonding enhancement	Intermittent or prolonged KMC, Post-bath KMC	↑ Cerebral oxygenation, ↑ neurobehavior, ↓ cortisol, ↓ maternal depression, ↑ bonding, ↑ breastfeeding	KMC is a cost-effective, evidence-based method supporting physiological and emotional stabilization in both infants and caregivers, with added neurodevelopmental benefits.
Cue-based and individualized developmental care	- Infant behavioral cues - Oral feeding readiness - Caregiver education	Cue-based feeding, NIDCAP, self-efficacy training	↓ time to full oral feeds, ↑ caregiver confidence, ↑ neurodevelopment at 8 years	Individualized care aligned with infant cues enhances feeding outcomes and long-term development. Increases caregiver competence and reduces NICU stress.
Environmental optimization	- Sensory regulation - Structured NICU design - Healing environments	Clustering care, SBU model, light/dark cycles, music therapy, maternal singing	↑ sleep, ↓ pain, ↓ infection, ↑ parental-infant bonding	Modifying NICU environments improves neurobehavior and physiological stability. Balanced sensory input is critical to optimizing development and emotional wellbeing.
Multisensory and psychosocial interventions	- Emotional bonding - Maternal mental health - Neurodevelopmental stimulation	Music therapy, maternal singing, caregiver interaction during care	↓ maternal anxiety, ↑ parental empowerment, ↑ infant regulation	Non-invasive multisensory inputs complement core care models by strengthening parent-infant connection and maternal wellbeing.
Contextual and implementation factors	- Cultural adaptation - Health system integration - Digital tools	Digital-FICare, mobile-based support, culturally tailored caregiver education	Improved scalability, engagement, and access in diverse NICU settings	Implementation success is highly dependent on cultural fit, resource availability, and institutional readiness. Digital health innovations offer promising pathways.

#### 3.4.1 Family integrated care (FICare): multidimensional benefits for infants and parents

Family Integrated Care (FICare) emerged as the most extensively evaluated approach, with studies consistently reporting its positive impact on neonatal outcomes and parental wellbeing across diverse cultural contexts such as Canada, China, Turkey, the Netherlands, and Finland (12, 21, 27, 28, 32). Improvements included increased breastfeeding rates, daily weight gain, reduced hospital stay, and enhanced discharge readiness. Furthermore, long-term developmental gains, particularly in self-regulation and socio-emotional domains, were observed at 18–24 months of corrected age (18, 28, 32). Psychosocial benefits were equally significant, with reduced parental stress (12) and improved maternal role attainment (28). Integration of mobile health tools enhanced parental engagement, reinforcing the scalability and digital adaptability of the FICare model (49).

#### 3.4.2 Kangaroo mother care (KMC): cost-effective and culturally resonant

KMC was reaffirmed as a low-cost, high-impact intervention with benefits extending across physiological, psychological, and developmental domains. Studies from India, Turkey, Egypt, and Brazil confirmed improvements in thermal regulation, oxygen saturation, neurobehavior, and breastfeeding initiation (13, 16, 29, 48). Neurophysiological mechanisms were evidenced by reduced stress biomarkers and improved perfusion index (17, 46). KMC also strengthened maternal-infant attachment, decreased maternal depression (35), and enhanced paternal involvement when both parents participated (37). The holistic nature of KMC, including its neuroprotective and emotional bonding effects, makes it particularly suitable for resource-limited settings.

#### 3.4.3 Cue-based and individualized developmental care: enhancing neurodevelopment

Developmentally supportive models such as cue-based feeding and the Newborn Individualized Developmental Care and Assessment Program (NIDCAP) demonstrated improved feeding performance, shorter time to full oral feeds, and better long-term cognitive and motor outcomes (39, 40). Educational interventions increased caregiver self-efficacy (34) and parental participation (42, 47). These findings emphasize the importance of synchronizing caregiving with infants’ behavioral cues to optimize neurodevelopment and promote caregiver confidence.

#### 3.4.4 Environmental modifications: from sensory protection to circadian health

Several studies explored how changes to NICU environments, such as clustering care (45), minimizing sensory overload (50), and adjusting light/dark exposure (51), impact outcomes. While single-family rooms and dim lighting were associated with better sleep and infection control (38), some studies (8) raised concerns about sensory deprivation affecting neurodevelopment. Others, such as Kostilainen et al. (30), showed that maternal singing and music therapy during kangaroo care improved maternal wellbeing and parent-infant interaction. However, caution is warranted regarding sensory deprivation risks, as single-family room models were associated with mixed developmental outcomes depending on implementation fidelity (8, 11).

#### 3.4.5 Multisensory and psychosocial interventions: promising adjuncts

Emerging evidence supports the use of integrative approaches such as music therapy (7) and maternal singing (30) to reduce maternal anxiety and improve emotional bonding. These interventions often complemented KMC and FICare, fostering parent-infant synchrony and promoting parental wellbeing.

#### 3.4.6 Contextual factors shaping implementation and sustainability

The implementation and sustainability of developmental care interventions across neonatal intensive care units (NICUs) were consistently shaped by contextual elements, including infrastructural capacity, caregiver literacy, cultural norms, and institutional readiness. In several studies, logistical constraints such as staffing limitations, crowded NICUs, and limited training infrastructure were reported barriers to consistent delivery of interventions like Family Integrated Care (FICare) and Kangaroo Mother Care (KMC) (27, 39). Programs that adopted participatory or co-designed models, such as those engaging both parents in caregiving roles were better able to adapt to these challenges. For instance, Yurdagül and Esenay (37) found that KMC administered by both parents improved physiological outcomes more effectively than mother-only interventions, suggesting a benefit to culturally inclusive practices.

In lower-resource contexts, studies such as those by Chaudhari et al. (29) in India and El-Farrash et al. (13) in Egypt showed that enhanced caregiver education and extended skin-to-skin contact significantly improved neonatal stability and neurobehavioral outcomes, despite constrained resources. Similarly, the use of simplified, scalable interventions, like cue-based feeding protocols (34, 40) demonstrated feasibility and effectiveness across both high- and middle-income settings. These findings support the importance of tailoring developmental care strategies to cultural norms and infrastructural realities to ensure both uptake and long-term integration within neonatal care systems.

## 4 Discussion

This systematic review synthesizes evidence from 35 studies examining a range of developmental care interventions for preterm infants, including family integrated care (FICare), kangaroo mother care (KMC), cue-based care, environmental modifications, and individualized neurodevelopmental support. The findings of this review reinforce and expand upon the work of Velasco Arias et al. (20), who conducted a scoping review of developmental-centered care in preterm newborns. Similar to their conclusions, our review confirms that developmental care interventions, particularly Family Integrated Care (FICare), Kangaroo Mother Care (KMC), and individualized neuroprotective strategies consistently yield positive outcomes across physiological, neurodevelopmental, and psychosocial domains. Both reviews highlight the importance of family engagement and environmental adaptations in improving care quality; however, this review provides deeper insights into the specific effectiveness of individual interventions such as cue-based care, neurobehavioral modulation, and digital integration within FICare frameworks. Whereas Velasco Arias et al. focused primarily on mapping existing intervention categories and theoretical principles, our findings contribute further granularity by evaluating outcomes based on study designs, population characteristics, and follow-up durations. Importantly, this review also addresses implementation science considerations, such as contextual barriers, parental literacy, and digital scalability which were only briefly noted in the previous scoping review. Moreover, while Velasco Arias et al. acknowledged the need for culturally sensitive care, our synthesis includes more recent evidence from LMIC settings and stresses the importance of tailored, resource-adaptive strategies for sustaining impact in diverse healthcare environments. Thus, our review not only confirms the foundational importance of developmental care principles identified by prior literature but also advances the field by detailing emerging implementation approaches and outcome standardization needs.

Our review reinforces findings from more recent studies such as van Veenendaal et al. (11) and Murphy et al. (27), who reported that Family Integrated Care (FICare) improves weight gain, shortens length of stay, and enhances parental participation in the NICU. Notably, our findings extend this evidence by illustrating the scalability of FICare through digital platforms and culturally contextualized models in upper-middle-income countries like China and Turkey (21). This broader applicability contrasts with earlier literature (27) that focused predominantly on high-income settings and did not account for digital innovations. In addition, our review confirms that the positive effects of FICare persist beyond discharge, with studies reporting improved cognitive and motor development at 18–24 months corrected age (18). This directly addresses previous critiques that developmental care literature focused excessively on short-term physiological outcomes.

Regarding Kangaroo Mother Care (KMC), recent syntheses (29) reaffirm its role in stabilizing cerebral hemodynamics and supporting neurobehavioral development, complementing the known mortality and hypothermia benefits. We also identified a novel emphasis on behavioral and neurodevelopmental outcomes such as pain attenuation and improved sleep which this outcomes previously underreported (52). Importantly, duration of exposure emerged as a critical factor, with longer KMC linked to improved neurodevelopmental trajectories (53), supporting the need for standardized protocols and sustained caregiver engagement. Cue-based and individualized developmental care, such as the Newborn Individualized Developmental Care and Assessment Program (NIDCAP) was shown to offer long-term developmental benefits across global contexts. While earlier studies such as Symington and Pinelli (54) established foundational outcomes, recent longitudinal studies confirm sustained cognitive and motor advantages through early childhood, including in non-Western settings.

Evidence regarding environmental interventions like Single Family Rooms (SFRs) remains mixed. While benefits such as infection control and enhanced bonding were evident (Lester et al., 2021), newer studies also reported concerns about reduced sensory stimuli potentially delaying language development. These dual outcomes reflect the architectural and developmental tension in NICU design, emphasizing the importance of a balanced sensory environment (43).

A key strength of this review is its focus on implementation science. Studies increasingly report that developmental care success depends not only on intervention fidelity but also on local readiness, staff training, parental health literacy, and sociocultural norms (34). This aligns with contemporary implementation frameworks such as the Consolidated Framework for Implementation Research (CFIR) and highlights a critical shift in the field from testing “if an intervention works” to understanding how and under what conditions it works (55).

### 4.1 Gaps and recommendations

Despite the growing body of evidence supporting developmental care interventions for preterm infants, several critical gaps remain evident across the literature. One prominent limitation is the fragmented nature of existing research, with most studies evaluating singular interventions such as kangaroo mother care (KMC) or family integrated care (FICare), rather than assessing the synergistic potential of multi-component or systems-based developmental care models (12, 21, 27). This narrow focus restricts understanding of how these approaches interact and complement each other in real-world clinical settings. Furthermore, there is substantial heterogeneity in the outcomes measured and the tools used across studies. While some employed standardized neurodevelopmental assessments like the Bayley Scales (32), others relied on observational checklists or institution-specific indicators, complicating efforts to synthesize findings and conduct meta-analyses.

Another critical gap lies in the geographic distribution of research. A majority of studies were conducted in high-income countries, particularly Canada, Turkey, and the Netherlands, with relatively few rigorous studies emerging from low- and middle-income countries (LMICs), despite the disproportionate burden of preterm births in these settings (13, 29, 48). The underrepresentation of LMIC contexts limits the global applicability of findings and raises questions about the feasibility of implementing certain interventions in resource-constrained environments. Compounding this issue is the scarcity of studies incorporating long-term follow-up beyond the neonatal period. Although a few longitudinal evaluations have demonstrated sustained benefits of interventions like NIDCAP and FICare on motor and cognitive outcomes into early childhood (18), most studies concluded at NICU discharge or within the first year of life, leaving the extended developmental trajectory largely unexplored.

A notable limitation of the current evidence base is the limited use of established implementation science frameworks to examine how developmental care interventions are adopted, integrated, and sustained within real-world NICU environments. Many of the included studies focused primarily on clinical outcomes without systematically evaluating critical implementation domains such as caregiver readiness, staff training requirements, organizational culture, cultural acceptability, and infrastructure constraints (20). These factors play a central role in determining whether an intervention that is effective under controlled conditions can be successfully scaled or embedded into routine practice. The absence of such analyses risks overlooking how interventions interact with the complexity of NICU workflows, multidisciplinary teams, and resource variability, particularly in low- and middle-income countries (LMICs).

Addressing these gaps requires a deliberate shift toward systems-oriented research designs that conceptualize developmental care as an integrated framework rather than as discrete practices. Future studies should systematically apply implementation science models such as the Consolidated Framework for Implementation Research (CFIR) or RE-AIM to assess feasibility, fidelity, contextual barriers, and sustainability over time. Mixed-methods and longitudinal designs are essential for capturing both clinical outcomes and implementation processes, including caregiver engagement, health worker adoption, and contextual adaptations (34). Applying CFIR would allow researchers to map how developmental care components interact with organizational culture, staffing models, caregiver readiness, and contextual constraints within NICUs. Likewise, the RE-AIM framework (Reach, Effectiveness, Adoption, Implementation, Maintenance) offers a structured lens for assessing how well developmental care interventions penetrate diverse populations, achieve clinical and developmental outcomes, and sustain their impact over time. Additionally, the i-PARIHS framework (integrated-Promoting Action on Research Implementation in Health Services) can guide evaluations of facilitation strategies and context-specific adaptations, which are vital in LMIC NICU settings where infrastructural and cultural variability may influence program uptake.

In addition, establishing core outcome sets and standardized tools would improve consistency and comparability across studies, enabling stronger cross-study inferences. Targeted investment is also needed to advance developmental care research in LMICs through community-engaged, culturally sensitive approaches that account for resource limitations and sociocultural norms. Emerging digital innovations, such as mobile health applications, tele-coaching platforms, and remote monitoring tools offer promising pathways for scaling interventions equitably. Evidence from early studies integrating these technologies within Kangaroo Mother Care (KMC) and Family Integrated Care (FICare) models demonstrates improvements in caregiver engagement, knowledge retention, and continuity of care. Expanding these technology-enabled strategies could enhance global access to developmental care and strengthen neonatal outcomes across diverse settings.

### 4.2 Limitations

Several limitations should be acknowledged in interpreting the findings of this review. First, although the search strategy was comprehensive and covered multiple databases, the review was restricted to studies published in English. This language restriction may have excluded relevant research from non-English-speaking regions, including low- and middle-income countries (LMICs), where innovative developmental care practices may be reported in local languages. Second, the substantial heterogeneity across the included studies—in terms of methodological design, intervention modalities, outcome measures, follow-up periods, and contextual factors—precluded the possibility of conducting a meta-analysis or performing direct quantitative comparisons. Consequently, the ability to draw robust generalizable estimates of effect is limited. Third, although both quantitative and qualitative evidence was incorporated, methodological quality varied considerably, particularly among observational and quasi-experimental studies, potentially influencing the strength and reliability of the conclusions. Fourth, publication bias remains a possibility; studies with positive or statistically significant findings are more likely to be published, and grey literature, conference proceedings, and non-indexed sources were not systematically screened. Lastly, while this review aimed to explore contextual and implementation-related dimensions of developmental care, reporting on these aspects was often limited or insufficiently detailed in the included studies, thereby constraining the depth of synthesis that could be achieved using implementation science or health systems frameworks.

## 5 Conclusion

This scoping review affirms the value of developmental care interventions in improving short- and long-term outcomes among preterm infants, with strong evidence supporting models such as Family Integrated Care (FICare), Kangaroo Mother Care (KMC), and individualized neurodevelopmental support. These interventions not only benefit physiological and neurodevelopmental outcomes but also enhance parental involvement, reduce stress, and support smoother transitions from NICU to home. Importantly, the findings underscore the role of contextual adaptation, digital integration, and culturally responsive approaches in enhancing implementation and sustainability, especially in LMICs where health system challenges are pronounced. Moving forward, research should prioritize systems-based evaluations, standardized outcome reporting, long-term follow-up, and implementation science approaches to advance equitable and scalable models of care for this vulnerable population.
