# Supplementary material for: Developmental care for preterm infants: a scoping review of interventions, outcomes, and implementation contexts
Source: Front Pediatr. 2026 Feb 5;14:1730571. doi: 10.3389/fped.2026.1730571 (PMC12916686; doi:10.3389/fped.2026.1730571)
Supplement: Supplementary file 1 [file Table1.docx]

MMAT berbeda per desain (RCT, non-RCT, kualitatif, mixed-methods, dsb.), format tabel berikut saya buat dengan kolom:

- No
- Authors (Year)
- Study Design
- MMAT Criterion 1
- MMAT Criterion 2
- MMAT Criterion 3
- MMAT Criterion 4
- MMAT Criterion 5
- Overall Rating

Keterangan singkat kriteria MMAT (RCT contoh):

- **C1:** Apakah randomisasi dilakukan dengan tepat?
- **C2:** Apakah alokasi tersembunyi?
- **C3:** Apakah kelompok awal sebanding?
- **C4:** Apakah penilaian buta terhadap hasil?
- **C5:** Apakah data hasil lengkap tanpa bias hilangnya data?

**Tabel. Penilaian Kualitas Metodologis Studi dengan MMAT 2018 (Hong et al., 2018)**

| **No** | **Authors (Year)** | **Study Design** | **C1** | **C2** | **C3** | **C4** | **C5** | **Overall Rating** |
| --- | --- | --- | --- | --- | --- | --- | --- | --- |
| **Randomized Controlled Trials (RCTs)** | | | | | | | | |
| 1 | Alferink et al. (2025) | Stepped-wedge cluster RCT | Yes | Yes | Yes | Yes | Yes | 5/5 |
| 2 | Arslan et al. (2024) | RCT | Yes | Yes | Yes | Yes | Yes | 5/5 |
| 3 | Çaka et al. (2023) | RCT | Yes | Yes | Yes | Yes | Yes | 5/5 |
| 4 | Cristóbal Cañadas et al. (2022) | RCT | Yes | Yes | Yes | Yes | Yes | 5/5 |
| 5 | El-Farrash et al. (2020) | RCT | Yes | Yes | Yes | Yes | Yes | 5/5 |
| 6 | Erduran & Yaman Sözbir (2023) | RCT | Yes | Yes | Yes | Yes | Yes | 5/5 |
| 7 | Wang et al. (2022) | RCT | Yes | Yes | Yes | Yes | Yes | 5/5 |
| 8 | Tiryaki et al. (2024) | RCT | Yes | Yes | Yes | Yes | Yes | 5/5 |
| **Non-randomized quantitative studies (non-RCT)** | | | | | | | | |
| 9 | Chaudhari et al. (2023) | Quasi-experimental | Yes | Yes | Yes | Can’t tell | Yes | 4/5 |
| 10 | Church et al. (2020) | Quasi-experimental | Yes | Yes | Can’t tell | Yes | Yes | 4/5 |
| 11 | Endo et al. (2021) | Quasi-experimental | Yes | Yes | Yes | Can’t tell | Yes | 4/5 |
| 12 | Hendy et al. (2022) | Quasi-experimental | Yes | Yes | Yes | Yes | Yes | 5/5 |
| 13 | Kucukoglu et al. (2021) | Quasi-experimental | Yes | Yes | Yes | Yes | Can’t tell | 4/5 |
| 14 | Samane et al. (2022) | Non-randomized trial | Yes | Yes | Yes | Can’t tell | Yes | 4/5 |
| 15 | Solanki & Bookseller (2023) | Comparative study | Yes | Yes | Yes | Yes | Yes | 5/5 |
| 16 | Souza-Vogler & Lima (2021) | Observational study | Yes | Yes | Yes | Can’t tell | Can’t tell | 3/5 |
| 17 | Thomas et al. (2021) | Quality improvement | Yes | Yes | Yes | Can’t tell | Yes | 4/5 |
| 18 | Veeraiah et al. (2023) | Pre-post | Yes | Yes | Yes | Yes | Can’t tell | 4/5 |
| 19 | Zahedpasha et al. (2021) | Non-randomized trial | Yes | Yes | Yes | Yes | Yes | 5/5 |
| **Mixed-methods** | | | | | | | | |
| 20 | Kostilainen et al. (2021) | Mixed-methods | Yes | Yes | Yes | Yes | Yes | 5/5 |
| 21 | McFadden et al. (2021) | Mixed-methods feasibility | Yes | Yes | Yes | Yes | Yes | 5/5 |
| **Quantitative descriptive / cohort / retrospective** | | | | | | | | |
| 22 | Fathi et al. (2022) | Descriptive study | Yes | Yes | Yes | Yes | Can’t tell | 4/5 |
| 23 | Liang et al. (2022) | Retrospective cohort | Yes | Yes | Yes | Yes | Yes | 5/5 |
| 24 | Moe et al. (2022) | Prospective cohort | Yes | Yes | Yes | Yes | Yes | 5/5 |
| 25 | Mohamed et al. (2023) | Prospective study | Yes | Yes | Yes | Yes | Yes | 5/5 |
| 26 | Moreno-Sanz et al. (2025) | Case-control | Yes | Yes | Yes | Yes | Yes | 5/5 |
| 27 | Murphy et al. (2021) | Cluster-RCT | Yes | Yes | Yes | Yes | Yes | 5/5 |
| 28 | O’Shea et al. (2023) | Longitudinal cohort | Yes | Yes | Yes | Yes | Yes | 5/5 |
| 29 | Pavlyshyn et al. (2021) | Prospective cohort | Yes | Yes | Yes | Yes | Yes | 5/5 |
| 30 | Rivera (2024) | Program development | Can’t tell | Yes | Yes | Yes | Can’t tell | 3/5 |
| 31 | Yang et al. (2021) | Preliminary comparative | Yes | Yes | Yes | Yes | Yes | 5/5 |
| 32 | van Veenendaal et al. (2020) | Retrospective cohort + mediation | Yes | Yes | Yes | Yes | Yes | 5/5 |
| 33 | Menke et al. (2021) | Pilot study | Yes | Yes | Yes | Yes | Yes | 5/5 |
| 34 | Yurdagül & Esenay (2023) | Crossover study | Yes | Yes | Yes | Yes | Yes | 5/5 |
| 35 | Jing et al. (2023) | Experimental | Yes | Yes | Yes | Yes | Yes | 5/5 |
