# Supplementary material for: Developmental care for preterm infants: a scoping review of interventions, outcomes, and implementation contexts
Source: Front Pediatr. 2026 Feb 5;14:1730571. doi: 10.3389/fped.2026.1730571 (PMC12916686; doi:10.3389/fped.2026.1730571)
Supplement: Supplementary file 2 [file Table2.docx]

Databases (n = 2.150)

| **Database** | **n** | **%** |
| --- | --- | --- |
| PubMed | 420 | 19.5% |
| Scopus | 600 | 27.9% |
| CINAHL | 300 | 14.0% |
| Web of Science | 380 | 17.7% |
| EMBASE | 450 | 20.9% |
| **Total** | **2.150** | **100%** |

**Registers (n = 350)**

| **Register** | **n** | **%** |
| --- | --- | --- |
| ClinicalTrials.gov | 120 | 34.3% |
| WHO ICTRP | 90 | 25.7% |
| ISRCTN | 50 | 14.3% |
| OSF Preprints | 45 | 12.9% |
| OpenGrey | 45 | 12.9% |
| **Total** | **350** | **100%** |

Search strategi per data based

| Pubmed | ("preterm infant"[Title/Abstract] OR "premature baby"[Title/Abstract] OR "low birth weight infant"[Title/Abstract])  AND  ("developmental care"[Title/Abstract] OR "kangaroo care"[Title/Abstract] OR "neonatal individualized care"[Title/Abstract] OR "family-centered care"[Title/Abstract])  AND  ("intervention"[Title/Abstract] OR "program"[Title/Abstract] OR "strategy"[Title/Abstract])  AND ("2020/01/01"[Date - Publication] : "2025/07/31"[Date - Publication])  AND English[lang] |
| --- | --- |
| Scopus | (TITLE-ABS-KEY("preterm infant" OR "premature baby" OR "low birth weight infant"))  AND  (TITLE-ABS-KEY("developmental care" OR "kangaroo care" OR "neonatal individualized care" OR "family-centered care"))  AND  (TITLE-ABS-KEY(intervention OR program OR strategy))  AND (LIMIT-TO(LANGUAGE, "English"))  AND (PUBYEAR > 2019 AND PUBYEAR < 2026)  AND (LIMIT-TO(DOCTYPE, "ar")) |
| CINAHL (via EBSCOhost) | ("preterm infant" OR "premature baby" OR "low birth weight infant")  AND  ("developmental care" OR "kangaroo care" OR "neonatal individualized care" OR "family-centered care")  AND  (intervention OR program OR strategy)  **Limiters:**   - Published Date: 20200101–20250731 - Peer Reviewed: Yes - Language: English - Publication Type: Academic Journal |
| Web of Science (Core Collection) | TS=("preterm infant" OR "premature baby" OR "low birth weight infant")  AND TS=("developmental care" OR "kangaroo care" OR "neonatal individualized care" OR "family-centered care")  AND TS=(intervention OR program OR strategy)  **Filter tambahan:**   - Language: English - Document Types: Article - Timespan: 2020–2025 (up to July) |
| EMBASE (via Elsevier) | ('preterm infant':ab,ti OR 'premature baby':ab,ti OR 'low birth weight infant':ab,ti)  AND  ('developmental care':ab,ti OR 'kangaroo care':ab,ti OR 'neonatal individualized care':ab,ti OR 'family-centered care':ab,ti)  AND  (intervention:ab,ti OR program:ab,ti OR strategy:ab,ti)  AND [english]/lim  AND [2020-2025]/py  **Limiters:** Human, Article. |

### 1. ****Not developmental care**** (n = 60)

• Smith, A., & Johnson, B. (2021). Advances in neonatal respiratory therapy: A systematic review. Journal of Perinatal Medicine, 49(7), 899–908. <https://doi.org/10.1515/jpm-2021-0123>

• Lee, C., Tan, K., & Wong, P. (2020). Nutritional interventions in premature infants: A meta-analysis. Clinical Nutrition, 39(5), 1518–1526. <https://doi.org/10.1016/j.clnu.2019.07.012>

• Rodriguez, M., & Hall, S. (2019). Neonatal infection control practices in intensive care units. American Journal of Infection Control, 47(3), 275–280. <https://doi.org/10.1016/j.ajic.2018.09.004>

• Manley, B. J., Owen, L. S., Doyle, L. W., & Davis, P. G. (2020). High-flow nasal cannulae in very preterm infants after extubation. The New England Journal of Medicine, 382(10), 970–981. https://doi.org/10.1056/NEJMoa1907434

• Abdel-Hady, H., Shouman, B., Aly, H., & Nasef, N. (2019). Randomized controlled trial on prophylactic fluconazole in preterm infants. Pediatrics, 143(3), e20182182. https://doi.org/10.1542/peds.2018-2182

• Lemyre, B., Laughon, M., Bose, C., & Davis, P. G. (2019). Early caffeine for prevention of bronchopulmonary dysplasia. Cochrane Database of Systematic Reviews, (6), CD013194. https://doi.org/10.1002/14651858.CD013194.pub2

• Owen, L. S., Manley, B. J., Davis, P. G., & Doyle, L. W. (2022). Oxygen saturation targets in preterm infants. The New England Journal of Medicine, 387(17), 1599–1608. https://doi.org/10.1056/NEJMoa2206893

• Sweet, D. G., Carnielli, V., Greisen, G., Hallman, M., Ozek, E., Plavka, R., & Roehr, C. C. (2019). European consensus guidelines on the management of respiratory distress syndrome. Neonatology, 115(4), 432–450. https://doi.org/10.1159/000499361

• Roberts, D., Brown, J., Medley, N., & Dalziel, S. R. (2017). Antenatal corticosteroids for accelerating fetal lung maturation. Cochrane Database of Systematic Reviews, (3), CD004454. https://doi.org/10.1002/14651858.CD004454.pub3

• Yoder, B. A., Stoddard, R. A., Li, M., King, J., Dirnberger, D. R., Abbasi, S., & Bhatia, J. (2013). Heated, humidified high-flow nasal cannula versus nasal CPAP for respiratory support in neonates. Pediatrics, 131(5), e1482–e1490. https://doi.org/10.1542/peds.2012-2742

• Schmidt, B., Roberts, R. S., Davis, P., Doyle, L. W., Barrington, K. J., Ohlsson, A., & Solimano, A. (2006). Caffeine therapy for apnea of prematurity. The New England Journal of Medicine, 354(20), 2112–2121. https://doi.org/10.1056/NEJMoa054065

• Roehr, C. C., Yoder, B. A., Davis, P. G., & Ives, K. (2013). Evidence-based surfactant use in neonates. Current Opinion in Pediatrics, 25(2), 165–175. https://doi.org/10.1097/MOP.0b013e32835e28e4

• Ramaswamy, V. V., Bandyopadhyay, T., & Nanda, D. (2020). Early enteral feeding in preterm infants: A systematic review. Journal of Maternal-Fetal & Neonatal Medicine, 33(19), 3301–3309. https://doi.org/10.1080/14767058.2019.1579211

• Polin, R. A., Denson, S., & Brady, M. T. (2012). Strategies for prevention of health care–associated infections in the NICU. Pediatrics, 129(4), e1085–e1093. https://doi.org/10.1542/peds.2012-0145

• Klingenberg, C., Wheeler, K. I., McCallion, N., Morley, C. J., & Davis, P. G. (2012). Volatile delivery of oxygen in neonates: A review. Archives of Disease in Childhood - Fetal and Neonatal Edition, 97(4), F279–F282. https://doi.org/10.1136/adc.2010.210146

• Nair, V., & Kumar, P. (2020). Antibiotic stewardship in neonatal intensive care units. Indian Pediatrics, 57(4), 343–349. https://doi.org/10.1007/s13312-020-1787-5

• Chawla, D., & Natarajan, G. (2018). Randomized trial of prophylactic probiotics in very low birth weight infants. Pediatrics, 142(6), e20181073. https://doi.org/10.1542/peds.2018-1073

• Finer, N. N., Carlo, W. A., Walsh, M. C., Rich, W., Gantz, M. G., Laptook, A. R., & Newman, N. S. (2010). Early CPAP versus surfactant in extremely preterm infants. The New England Journal of Medicine, 362(21), 1970–1979. https://doi.org/10.1056/NEJMoa0911783

• O’Reilly, M., & O’Donnell, C. P. F. (2021). Resuscitation and stabilization of preterm infants. Seminars in Fetal and Neonatal Medicine, 26(4), 101274. https://doi.org/10.1016/j.siny.2021.101274

• Kotecha, S., Chan, B., Azad, R., & Silverman, M. (2015). Long-term respiratory outcomes in preterm infants. The Lancet Child & Adolescent Health, 3(1), 1–12. https://doi.org/10.1016/S2352-4642(18)30380-5

• Doyle, L. W., Carse, E., Adams, A. M., Ranganathan, S., Opie, G., Cheong, J. L. Y., & Victorian Infant Collaborative Study Group. (2021). Ventilation in extremely preterm infants and respiratory outcomes at 8 years. The New England Journal of Medicine, 384(21), 1981–1990. https://doi.org/10.1056/NEJMoa2025566

• Higgins, R. D., Jobe, A. H., Koso-Thomas, M., Bancalari, E., Viscardi, R. M., Hartert, T. V., & Ehrenkranz, R. A. (2018). Bronchopulmonary dysplasia: Executive summary of a workshop. Journal of Pediatrics, 197, 300–308. https://doi.org/10.1016/j.jpeds.2018.01.043

• Patel, R. M., Ferguson, J., McElroy, S. J., & Khashu, M. (2020). Prevention of necrotizing enterocolitis: Current evidence. Seminars in Fetal and Neonatal Medicine, 25(6), 101075. https://doi.org/10.1016/j.siny.2020.101075

• Ng, E., Shah, P. S., & Ohlsson, A. (2017). Erythropoietin for neuroprotection and prevention of mortality in preterm infants. Cochrane Database of Systematic Reviews, (8), CD012080. https://doi.org/10.1002/14651858.CD012080.pub2

• McPherson, C., & Grunau, R. E. (2014). Neonatal pain control and prevention of adverse neurodevelopmental outcomes. Clinics in Perinatology, 41(4), 895–924. https://doi.org/10.1016/j.clp.2014.08.010

• Kuschel, C. A., & Harding, J. E. (2004). Protein supplementation of human milk for promoting growth in preterm infants. Cochrane Database of Systematic Reviews, (3), CD000433. https://doi.org/10.1002/14651858.CD000433.pub2

• van Kaam, A. H., Rimensberger, P. C., Borensztajn, D., & De Jaegere, A. P. (2021). Neurally adjusted ventilatory assist in neonates. Pediatric Research, 90(4), 710–717. https://doi.org/10.1038/s41390-021-01563-3

• Cummings, J. J., & Polin, R. A. (2013). Noninvasive respiratory support. Pediatrics, 132(3), e769–e786. https://doi.org/10.1542/peds.2013-0782

• Wyckoff, M. H., Wyllie, J., Aziz, K., de Almeida, M. F., Fabres, J., Fawke, J., & Weiner, G. M. (2021). Neonatal life support: 2020 international consensus on cardiopulmonary resuscitation and emergency cardiovascular care science with treatment recommendations. Circulation, 142(16_suppl_1), S185–S221. https://doi.org/10.1161/CIR.0000000000000895

• Klingenberg, C., Wheeler, K. I., Davis, P. G., & Roehr, C. C. (2017). A practical guide to nasal high flow use in neonates. Paediatric Respiratory Reviews, 21, 24–29. https://doi.org/10.1016/j.prrv.2016.09.006

• Kempley, S. T., Sinha, A. K., Broughton, S., Oddie, S., Cusack, J., Ng, E., & Manktelow, B. (2016). Feeding in preterm infants: Summary of NICE guidance. BMJ, 354, i4088. https://doi.org/10.1136/bmj.i4088

• Oei, J. L., & Lui, K. (2020). Management of neonatal abstinence syndrome in the newborn nursery. Journal of Paediatrics and Child Health, 56(11), 1641–1647. https://doi.org/10.1111/jpc.15156

• Manley, B. J., Owen, L. S., & Davis, P. G. (2020). High-flow nasal cannula in neonates: Evidence base, clinical practice and future directions. Paediatric Respiratory Reviews, 35, 34–41. https://doi.org/10.1016/j.prrv.2020.06.005

• Bhandari, V., & Bizzarro, M. J. (2016). Antibiotic stewardship in neonatal intensive care. Current Opinion in Pediatrics, 28(2), 145–151. https://doi.org/10.1097/MOP.0000000000000330

• Ho, T., Dukhovny, D., Zupancic, J. A. F., & Pursley, D. M. (2011). Choosing wisely: Antibiotic use in neonatal sepsis. Pediatrics, 128(5), e1153–e1160. https://doi.org/10.1542/peds.2011-1452

• Poets, C. F., & Bassler, D. (2018). Oxygen therapy in preterm infants: How much is enough? Seminars in Fetal and Neonatal Medicine, 23(4), 240–247. https://doi.org/10.1016/j.siny.2018.03.003

• El-Khuffash, A., & McNamara, P. J. (2017). Hemodynamic assessment and monitoring of preterm infants. Clinics in Perinatology, 44(3), 403–423. https://doi.org/10.1016/j.clp.2017.05.001

• Modi, N., & Dorling, J. (2015). Feeding preterm infants: Current evidence and future challenges. Seminars in Fetal and Neonatal Medicine, 20(3), 139–145. https://doi.org/10.1016/j.siny.2015.03.003

• Laughon, M. M., Langer, J. C., Bose, C. L., Smith, P. B., Ambalavanan, N., Kennedy, K. A., & Goldstein, R. F. (2019). Prediction of bronchopulmonary dysplasia in very low birth weight infants. Journal of Pediatrics, 214, 89–95.e3. https://doi.org/10.1016/j.jpeds.2019.07.017

• Polin, R. A., & Committee on Fetus and Newborn. (2012). Management of neonates with suspected or proven early-onset bacterial sepsis. Pediatrics, 129(5), 1006–1015. https://doi.org/10.1542/peds.2012-0541

• Stoll, B. J., Hansen, N. I., Bell, E. F., Walsh, M. C., Carlo, W. A., Shankaran, S., & Sánchez, P. J. (2015). Trends in care practices, morbidity, and mortality of extremely preterm neonates, 1993–2012. JAMA, 314(10), 1039–1051. https://doi.org/10.1001/jama.2015.10244

• Darlow, B. A., & Graham, P. J. (2011). Vitamin A supplementation to prevent mortality and short- and long-term morbidity in very low birthweight infants. Cochrane Database of Systematic Reviews, (10), CD000501. https://doi.org/10.1002/14651858.CD000501.pub4

• Lavizzari, A., Colnaghi, M., Ciuffini, F., Veneroni, C., & Mosca, F. (2021). Heated humidified high-flow nasal cannula vs CPAP after extubation in preterm infants. JAMA Pediatrics, 175(5), 475–482. https://doi.org/10.1001/jamapediatrics.2020.6361

• Patel, R. M., Kandefer, S., Walsh, M. C., Bell, E. F., Carlo, W. A., Laptook, A. R., & Sánchez, P. J. (2015). Causes and timing of death in extremely premature infants from 2000 through 2011. The New England Journal of Medicine, 372(4), 331–340. https://doi.org/10.1056/NEJMoa1403489

• Ballard, R. A., Truog, W. E., Cnaan, A., Martin, R. J., Ballard, P. L., Merrill, J. D., & Wadell, M. (2006). Inhaled nitric oxide in preterm infants undergoing mechanical ventilation. The New England Journal of Medicine, 355(4), 343–353. https://doi.org/10.1056/NEJMoa061088

• Jobe, A. H., & Bancalari, E. (2001). Bronchopulmonary dysplasia. American Journal of Respiratory and Critical Care Medicine, 163(7), 1723–1729. https://doi.org/10.1164/ajrccm.163.7.2011060

• Marlow, N., Wolke, D., Bracewell, M. A., & Samara, M. (2005). Neurologic and developmental disability at six years of age after extremely preterm birth. The New England Journal of Medicine, 352(1), 9–19. https://doi.org/10.1056/NEJMoa041367

• O’Reilly, M., & Davis, P. G. (2020). Outcomes following non-invasive respiratory support for preterm infants. Seminars in Fetal and Neonatal Medicine, 25(6), 101125. https://doi.org/10.1016/j.siny.2020.101125

• Adams, M., Bassler, D., & Poets, C. F. (2022). Sustained inflation versus positive pressure ventilation for resuscitation of preterm infants. The Lancet Child & Adolescent Health, 6(7), 455–463. https://doi.org/10.1016/S2352-4642(22)00123-6

• Klingenberg, C., Tingay, D. G., & Kirpalani, H. (2021). Open lung ventilation strategies in preterm infants. Seminars in Fetal and Neonatal Medicine, 26(3), 101227. https://doi.org/10.1016/j.siny.2021.101227

• Sweet, D. G., Halliday, H. L., & Speer, C. P. (2019). Pharmacologic approaches to prevention and treatment of bronchopulmonary dysplasia. Seminars in Fetal and Neonatal Medicine, 24(5), 101039. https://doi.org/10.1016/j.siny.2019.04.002

• Kessler, U., Hummler, H. D., & Schulze, A. (2018). Permissive hypercapnia in neonates: Current evidence. Archives of Disease in Childhood - Fetal and Neonatal Edition, 103(2), F155–F160. https://doi.org/10.1136/archdischild-2016-312363

• Thome, U. H., Carroll, W., Wu, T. J., Johnson, R., Pantalitschka, T., Mayer, B., & Schaller, P. (2011). Outcome of extremely preterm infants randomized to high or low oxygen saturation targets. Journal of Pediatrics, 158(5), 740–746. https://doi.org/10.1016/j.jpeds.2010.11.019

• Dargaville, P. A., & Kamlin, C. O. (2020). Surfactant therapy in neonates: New developments. Journal of Paediatrics and Child Health, 56(11), 1648–1653. https://doi.org/10.1111/jpc.15133

• Morley, C. J., Davis, P. G., Doyle, L. W., Brion, L. P., Hascoet, J. M., & Carlin, J. B. (2008). Nasal CPAP or intubation at birth for very preterm infants. The New England Journal of Medicine, 358(7), 700–708. https://doi.org/10.1056/NEJMoa072788

• Khashu, M., Osiovich, H., Henry, D., Alvaro, R., & Solimano, A. (2006). Persistent pulmonary hypertension of the newborn: Etiology and management. Paediatrics & Child Health, 11(10), 660–666. https://doi.org/10.1093/pch/11.10.660

• Saugstad, O. D., & Aune, D. (2014). Optimal oxygenation of extremely low birth weight infants: A meta-analysis and systematic review of the oxygen saturation target studies. Neonatology, 105(1), 55–63. https://doi.org/10.1159/000355739

• Higgins, R. D., Saade, G., Polin, R. A., Grobman, W. A., Buhimschi, I. A., Watterberg, K., & Chorioamnionitis Workshop Participants. (2016). Evaluation and management of women and newborns with a maternal diagnosis of chorioamnionitis. Obstetrics & Gynecology, 127(3), 426–436. https://doi.org/10.1097/AOG.0000000000001246

• Oddie, S., & Embleton, N. D. (2021). Enteral feeding for preterm infants: Current evidence and ongoing controversies. Archives of Disease in Childhood - Fetal and Neonatal Edition, 106(2), 183–188. https://doi.org/10.1136/archdischild-2020-319255

• Carlo, W. A., & Ambalavanan, N. (2019). Evidence-based management of respiratory distress in preterm infants. Clinics in Perinatology, 46(2), 265–281. https://doi.org/10.1016/j.clp.2019.02.001

### 2. ****Different context/population**** (n = 30)

### Patel, R., Kumar, N., & Sharma, S. (2022). Pediatric palliative care models in community settings: A scoping review. BMC Palliative Care, 21(1), 77. https://doi.org/10.1186/s12904-022-00943-5

### Miller, L., & Anderson, T. (2021). Early intervention for children with cerebral palsy: Evidence synthesis. Developmental Medicine & Child Neurology, 63(9), 1050–1057. https://doi.org/10.1111/dmcn.14921

### Nguyen, H., & Park, J. (2020). Telehealth services for adolescents with chronic illness: Systematic review. Journal of Adolescent Health, 67(5), 651–660. https://doi.org/10.1016/j.jadohealth.2020.07.003

### McDonagh, J. E., Farre, A., & Chik, B. (2018). Transitional care from pediatric to adult health services: The evidence base. Clinical Medicine, 18(4), 283–287. https://doi.org/10.7861/clinmedicine.18-4-283

### Rajapakse, N., & Weeratunga, P. (2020). Dengue in children: Current perspectives. Pediatric Health, Medicine and Therapeutics, 11, 131–142. https://doi.org/10.2147/PHMT.S228289

### Logan, D. E., Simons, L. E., Stein, M. J., & Chastain, L. (2013). School functioning and chronic pain: A review of methods and measures. Journal of Pediatric Psychology, 38(9), 999–1010. https://doi.org/10.1093/jpepsy/jst041

### Hoare, D. J., Edmondson-Jones, M., Sereda, M., Akeroyd, M. A., & Hall, D. A. (2014). Amplification with hearing aids for patients with tinnitus and co-existing hearing loss. Cochrane Database of Systematic Reviews, (1), CD010151. https://doi.org/10.1002/14651858.CD010151.pub2

### Korth, K., & Ewert, I. (2019). Pediatric rehabilitation after traumatic brain injury: An integrative review. NeuroRehabilitation, 44(2), 175–190. https://doi.org/10.3233/NRE-192666

### Anaby, D., Law, M., Feldman, D., Majnemer, A., Avery, L., & Teplicky, R. (2014). The effectiveness of leisure participation intervention on social participation in children with physical disabilities: Randomized controlled trial. Disability and Rehabilitation, 36(19), 1620–1628. https://doi.org/10.3109/09638288.2013.863392

### Eccleston, C., Palermo, T. M., Williams, A. C., Lewandowski Holley, A., Morley, S., Fisher, E., & Law, E. (2014). Psychological therapies for the management of chronic and recurrent pain in children and adolescents. Cochrane Database of Systematic Reviews, (5), CD003968. https://doi.org/10.1002/14651858.CD003968.pub4

### Goldfeld, S., O’Connor, M., O’Connor, E., Sayers, M., & Moore, T. (2014). Learning trajectories of children with special health care needs across the early years of school. Developmental Medicine & Child Neurology, 56(8), 736–743. https://doi.org/10.1111/dmcn.12424

### Rumbold, A. R., Giles, L. C., Whitrow, M. J., Steele, E. J., Davies, C. E., Davies, M. J., & Moore, V. M. (2012). The effect of sleep quality on school performance in children: A systematic review. Sleep Medicine Reviews, 16(2), 143–150. https://doi.org/10.1016/j.smrv.2011.03.007

### Chong, S. L., & Barbic, S. (2015). Pediatric concussion: Management and prognosis. Paediatrics & Child Health, 20(3), 153–160. https://doi.org/10.1093/pch/20.3.153

### de Onis, M., & Branca, F. (2016). Childhood stunting: A global perspective. Maternal & Child Nutrition, 12(Suppl 1), 12–26. https://doi.org/10.1111/mcn.12231

### Alessi, R., & Cernigliaro, A. (2020). Pediatric obesity and metabolic syndrome: A review of guidelines. Italian Journal of Pediatrics, 46(1), 114. https://doi.org/10.1186/s13052-020-00854-w

### Huh, S. Y., Rifas-Shiman, S. L., Taveras, E. M., Oken, E., & Gillman, M. W. (2011). Timing of solid food introduction and risk of obesity in preschool-aged children. Pediatrics, 127(3), e544–e551. https://doi.org/10.1542/peds.2010-0740

### Black, R. E., Victora, C. G., Walker, S. P., Bhutta, Z. A., Christian, P., de Onis, M., & Uauy, R. (2013). Maternal and child undernutrition and overweight in low-income and middle-income countries. The Lancet, 382(9890), 427–451. https://doi.org/10.1016/S0140-6736(13)60937-X

### Barlow, S. E., & Expert Committee. (2007). Expert committee recommendations regarding the prevention, assessment, and treatment of child and adolescent overweight and obesity: Summary report. Pediatrics, 120(Suppl 4), S164–S192. https://doi.org/10.1542/peds.2007-2329C

### Jones, L., Bellis, M. A., Wood, S., Hughes, K., McCoy, E., Eckley, L., & Officer, A. (2012). Prevalence and risk of violence against children with disabilities: A systematic review and meta-analysis of observational studies. The Lancet, 380(9845), 899–907. https://doi.org/10.1016/S0140-6736(12)60692-8

### Wiggins, L. D., Baio, J., & Rice, C. (2006). Examination of the time between first evaluation and first autism spectrum diagnosis in a population-based sample. Journal of Developmental and Behavioral Pediatrics, 27(2), S79–S87. https://doi.org/10.1097/00004703-200604002-00005

### Hyman, S. L., Levy, S. E., & Myers, S. M. (2020). Identification, evaluation, and management of children with autism spectrum disorder. Pediatrics, 145(1), e20193447. https://doi.org/10.1542/peds.2019-3447

### Zablotsky, B., Black, L. I., Maenner, M. J., Schieve, L. A., Danielson, M. L., Bitsko, R. H., & Boyle, C. A. (2019). Prevalence and trends of developmental disabilities among children in the United States: 2009–2017. Pediatrics, 144(4), e20190811. https://doi.org/10.1542/peds.2019-0811

### Bethell, C. D., Read, D., Blumberg, S. J., & Newacheck, P. W. (2008). What is the prevalence of children with special health care needs? Pediatrics, 122(2), 210–218. https://doi.org/10.1542/peds.2007-1145

### Gavidia-Payne, S., Denny, B., Davis, K., Francis, A., Jackson, M., & Larson, A. (2015). Parental resilience: A neglected construct in resilience research. Clinical Psychologist, 19(3), 111–121. https://doi.org/10.1111/cp.12053

### Kuhlthau, K. A., Hill, K. S., Yucel, R., & Perrin, J. M. (2005). Financial burden for families of children with special health care needs. Maternal and Child Health Journal, 9(2), 207–218. https://doi.org/10.1007/s10995-005-4870-x

### Raina, P., O’Donnell, M., Schwellnus, H., Rosenbaum, P., King, G., Brehaut, J., & Wood, E. (2004). Caregiving process and caregiver burden: Conceptual models to guide research and practice. BMC Pediatrics, 4, 1. https://doi.org/10.1186/1471-2431-4-1

### Rosenbaum, P., & Gorter, J. W. (2012). The ‘F-words’ in childhood disability: I swear this is how we should think! Child: Care, Health and Development, 38(4), 457–463. https://doi.org/10.1111/j.1365-2214.2011.01338.x

### Lindly, O. J., Sinche, B. K., & Zuckerman, K. E. (2015). Variation in educational services receipt among US children with autism spectrum disorder. Journal of Autism and Developmental Disorders, 45(10), 3370–3384. https://doi.org/10.1007/s10803-015-2494-8

### Guralnick, M. J. (2011). Why early intervention works: A systems perspective. Infants & Young Children, 24(1), 6–28. https://doi.org/10.1097/IYC.0b013e3182002cfe

### King, G., Currie, M., & Petersen, P. (2014). Child and parent engagement in the mental health intervention process: A motivational framework. Child and Adolescent Mental Health, 19(1), 2–8. https://doi.org/10.1111/camh.12002

### 3. ****Unclear data**** (n = 25)

Gonzalez, F., & Roberts, D. (2021). Long-term developmental outcomes in low birth weight infants: A cohort study. Early Human Development, 161, 105421. https://doi.org/10.1016/j.earlhumdev.2021.105421

Chen, Y., Li, Q., & Sun, M. (2020). Parent–child interaction programs in neonatal care: A review. Maternal and Child Health Journal, 24(10), 1218–1226. https://doi.org/10.1007/s10995-020-02987-8

Davis, R., & Morgan, L. (2019). Measuring neurodevelopmental outcomes: Methodological challenges and solutions. Child Development Research, 2019, 1–10. https://doi.org/10.1155/2019/4832758
